# “No more a child, not yet an adult”: studying social cognition in adolescence

**DOI:** 10.3389/fpsyg.2015.01011

**Published:** 2015-08-21

**Authors:** Adelina Brizio, Ilaria Gabbatore, Maurizio Tirassa, Francesca M. Bosco

**Affiliations:** ^1^Department of Psychology and Center for Cognitive Science, University of Turin, Turin, Italy; ^2^Faculty of Communication Science, Università della Svizzera Italiana, Lugano, Switzerland; ^3^Faculty of Humanities, Child Language Research Center, University of Oulu, Oulu, Finland; ^4^Department of Psychology and Neuroscience Institute of Turin, Turin, Italy

**Keywords:** adolescence, social cognition, theory of mind, mindreading, metacognition, self-reflection, development

## Abstract

There are several reasons why adolescence is interesting. It is in this phase that an individual finds herself fully facing the external world: basically equipped with the kind of social cognition that s/he has acquired at home, at school and through the media during childhood, s/he has now to meet a host of other, diverse views of what “reasonable,” “appropriate,” or “expected” courses of thought and emotions are, in the wild with friends and peers, romantic or sexual partners, teachers and employers, and the society at large. Furthermore, she is also expected, both at home and in the external world, to have a wholly new degree of control over such courses. While the idea that the development of social cognition still progresses after infancy (and possibly throughout the life span) is clearly gaining consensus in the field, the literature building on it is still scarce. One of the reasons for this probably is that most tests used to study it focus on its basic component, namely theory of mind, and have been mostly devised for us with children; therefore, they are not suitable to deal with the hugely increasing complexity of social and mental life during adolescence and adulthood. Starting from a review of the literature available, we will argue that the development of social cognition should be viewed as a largely yet-to-be-understood mix of biological and cultural factors. While it is widely agreed upon that the very initial manifestations of social life in the newborn are largely driven by an innate engine with which all humans are equally endowed, it is also evident that each culture, and each individual within it, develops specific adult versions of social cognition.

## Introduction

There are several reasons why adolescence—and, more to the point, social life and social cognition in adolescence—is scientifically interesting. It is during this period that an individual finds herself^1^ fully facing the external world: basically equipped with the social competences that she has acquired at home, at school and through the media during childhood, she has now to meet a host of other, views of what “reasonable,” “appropriate,” or “expected” courses of thought and emotions are, *in the wild* with friends and peers, romantic or sexual partners, teachers and employers, and the society at large. Furthermore, she is also expected, both at home and in the external world, to have a new degree of control over such courses.

Substantially analogous considerations hold when social cognition is viewed in its reflective aspects, that is as a means of self-knowledge. Again, adolescence is a crucial phase in the development of an individual’s understanding of herself, of her own feelings and desires, her own ways of reasoning, her own reactions to external as well as internal situations, etc. And, again, both the need and the actual ability to control these courses of thought and emotions change greatly during this period.

However, the literature on social cognition in adolescence is scarce and scattered, particularly if compared with other ages of life, like infancy and childhood, or other domains of cognition: few empirical studies are available and there is no unitary theoretical framework within which to understand them.

Yet, a better understanding of adolescence would be crucial, if only because a quantitatively impressive part of human kind currently falls within such period. The present generation of people aged 10–24 years is the largest in history: at 1.8 billion, it comprises a quarter of the world’s population. Nearly 90% of them live in low- or middle-income countries where, due to higher fertility rates, they constitute a far greater proportion of the population than in high-income countries (WHO, 2009).

Still another reason of interest is that several disorders—first and foremost, schizophrenia or substance abuse, but also mood and anxiety-related dysfunctions—have their onset or witness an increase during adolescence and early adulthood.

This makes it all the more interesting to understand what social cognition is in this phase of life, at least within the widely diffused theoretical framework that views a crucial aspect of social cognition, namely *theory of mind (ToM)*, as crucially involved in these disorders (Frith, 1992; for reviews, see Bosco et al., 2009a, and respectively, Bosco et al., 2014a).

This paper will discuss social cognition in adolescence. We will include both a review of the literature available and a theoretical discussion. We are aware that adolescence is, at least in part, a social construction, and that its features may vary between different sociocultural and historical therefore, we will do our best to keep our arguments on a sufficiently general level to allow for these differences. Yet, we are also aware that we are Western researchers, like most of our peers, and that this puts inescapable biases in our analyses.

## From Childhood to Adolescence

The social world is the most important realm of interaction of human beings, that within which we spend the whole of our lives. Even when we are alone with ourselves, be it at home or during a walk in the mountains, we are immersed in an environment which is more or less completely made of social artifacts and products; even more crucially, our thoughts and feelings, as well as our actions, are largely shaped by (and generally aimed at) the social world (Clancey, 1997).

Unsurprisingly, a great deal of effort has been devoted within the psychological sciences to investigating into the nature of human social cognition, its evolution in the species and its development in the individual.

As far as phylogeny is concerned, starting from pioneering research on so-called “Machiavellian intelligence” (e.g., Humphrey, 1976; Byrne and Whiten, 1985; Dunbar, 1993), it has been claimed that social cognition is at the very root of the particular evolution of the primates’ mind. Technology and a sophisticated use of artifacts would have played less important a role, at least until the appearance of the first hominins, since when the evolution of material cognition appears to have paralleled a further evolution of social cognition. The social life of humans, like that of the other great apes, is highly complex: they form long-term social relationship with others, understand the social relationship among third parties, and recognize that the actions of individuals are driven by their goals and by their perception of the situation (Tomasello and Vaish, 2013).

The phrase *social cognition* generally refers to “the various psychological processes that enable individuals to take advantage of being part of a social group” (Frith, 2008, p. 2033). It is crucial to put an emphasis on words like *cognition* and phrases like *to make sense*, which allow to keep social cognition proper as distinct from the mere influence that an individual’s behavior may or may not have on the behavior of other individuals (Bara and Tirassa, 2010), something which instead is universal in animals and even in plants.

An important facet of social cognition in primates is the ability to understand the mental states of other individuals, including their intentions, desires and beliefs, i.e., what is called *ToM*, *mentalizing*, or *folk psychology* (Davies and Stone, 1995; Nichols and Stich, 2003; Goldman, 2006; Blakemore et al., 2007; Hutto et al., 2011). Together with other sophisticated cognitive competences like social emotional processing (Burnett et al., 2009), this capacity enables an individual to understand, explain and predict another individual’s actions and thus also allows for the negotiation of complex interpersonal decisions (Crone, 2013). Social cognitive processes include also basic perceptual processes such as face processing (Farroni et al., 2005), biological motion detection (Pelphrey and Carter, 2008), and joint attention (Carpenter et al., 1998).

It is not completely understood if, or to what extent, this particular type of social cognition belongs to species other than ours (Heyes, 1998): it appears, however, that ToM-like competences are more widely diffused among primates than what is commonly thought (Tomasello, 2014). What can safely be said is that, while the social life of great apes is mainly about competition, human societies are vastly more and distinctively structured around and for cooperation (Tomasello and Vaish, 2013), which of course founds on the further evolution of peculiar cognitive competences, among which our special kind of social cognition. In a word, the social life of humans is largely a matter of *intersubjectivity* or *sharedness* (Premack and Premack, 1994; Tirassa, 1999; Tirassa and Bosco, 2008).

As regards ontogeny, there is now a remarkable amount of empirical literature in psychology describing the first years of development of social cognition and mindreading. This body of literature is far from yielding a univocal sense of what a child’s social cognition is or how it develops: rather, it appears to be a multidimensional, highly complex patchwork of different subareas, different theoretical or empirical approaches, and, necessarily, different results.

Most proposals in this area subscribe to a common philosophical framework defined by a set of core assumptions, namely, as stated above, that the primary function of social cognition is to predict, explain, and control the actions of the others, which is made possible by the attribution, and hence the representation, of their mental states. The big issue is whether such attributions are achieved by means of theoretical inference, simulative analogy, or a bit of both (Gallagher and Hutto, 2008; Hutto et al., 2011).

Furthermore, as Hutto et al. (2011) argue, most theories tacitly assume that human adults entertain a fully developed mindreading; consequently, the main question concerns the extent to which the mentalizing abilities of infants (if and when they indeed have any) might compare to those of the adult. However, there is a substantial lack of cognitive models of mindreading in adults (see the review in Apperly, 2013) or of what the phrase *a fully developed mindreading* could precisely mean.

Gallagher (2006) notes that ToM approaches to the explanation of how we come to understand others typically are abstract (third-person when they need to be second-person), mentalistic (starting with the supposition that there are things like minds, beliefs, desires that we have no access to in others, and sometimes even in ourselves), and biased toward theoretical reason (when practical, situated reason is a better way to go: see also Bosco et al., 2009b). Overly intellectualizing what is involved in our basic encounters with others, they tend to forget emotion and our ability to read it not in the minds of others, but on their faces, as well as in their gestures and expressive movements. Yet, if the basic ontology of human social competence is the same from the very beginning of mental life (as has been claimed, e.g., by Tirassa et al., 2006a,b), a more situated, more embodied approach to social cognition should be developed, allowing the more rationally sophisticated abilities to be a precious tool that comes into play when it is necessary rather than the only nature of human intersubjectivity.

These problems notwithstanding, there can be little doubt that crucial advancements in our understanding of the ontogeny of social cognition have been achieved in the last few decades from which knowledge is likely to proceed further, though probably still without a unitary theoretical framework in the foreseeable future.

Much less is known about the ways and directions in which social cognition develops after infancy: despite the increasing interest in social cognition beyond childhood (e.g., Valle et al., 2015) knowledge of how it works in the adult or possibly decays in the elderly still is scarce and scattered. There are several possible explanations for this situation (Dumontheil et al., 2010). Firstly, the tasks that have been used to test ToM in early development are not appropriate for testing older children and adolescents. Since most ToM tasks are passed by 5-years-olds, ceiling effects might be obscuring the observation of any further development. Secondly, tasks typically enquire directly children’s representations of another person’s mental states; they do not tap into how ToM is used to drive decisions and actions in everyday life.

When a situated framework is adopted, further problems pop up. One is that only one-to-one interactions are normally studied, and even these are framed in terms of a subject who is asked to observe and explain another individual’s behavior, rather than truly encountering him; that is, only a third-person perspective, instead of a second-person one, is adopted in practice. Another problem is that only real-time (vs. retrospective), status-free, culture-free interactions are taken into account, thus obliterating most of the complexities of real social life. Not only does the study of ToM in adolescence and early adulthood constitute a methodological challenge, insofar as it requires the creation of new empirical tasks fit to capture age differences (Henry et al., 2013; Moran, 2013; Valle et al., 2015): it also calls for a radical enrichment of the underlying theoretical frameworks.

Still another problem is that the ToM tasks that are normally used with children tend to impose rigid requirements on what the “right” answer is. This might be a reasonable choice, insofar as it can be assumed that all children—or, at least, all children of Western heritage—will basically follow the same developmental trajectories. It is less obvious, however, that the same may be the case of adults. Here, it might be said, “mindreader is as mindreader does”: there is no need to assume that a plateau should exist as the final state of the ontogeny of social cognition, and even less to assume that such plateau should be the same for all individuals in all historical contexts.

In line with at least part of the current literature (e.g., Apperly, 2012; Blakemore, 2012; Bosco et al., 2014b), our stance is that the ontogeny of social cognition does not end with childhood; instead, it continues through adolescence and the different ages of adulthood, as biological, social, cultural, educational, autobiographical, reflective, and retrospective changes accrue and become ever more intertwined and stratified.

The whole issue is further complicated by the fact that the psychological literature on adolescence in its turn offers an overwhelmingly ample (and still growing), but fractured, picture. This makes it difficult to achieve a deeper and coherent understanding of it (Moshman, 2005).

In principle, and roughly stated, there can exist three possible frameworks for understanding adolescence, namely as the exit from childhood, the entrance into adulthood, or a distinct stage of human life, a bridge between what comes before and after it but (comparatively) autonomous with respect to both. While each of these views has its merit, we argue that the second is preferable: adolescence as the beginning of adulthood (in agreement with Moshman, 2005).

Of course, this is not meant to imply that there can exist a divide between childhood and adolescence/adulthood: instead, the adoption of a life-course perspective promotes understanding that the factors affecting the individual during childhood can cumulatively affect her as an adolescent and an adult. At the same time, both normative and maladaptive patterns during adolescence shape future trajectories (Sawyer et al., 2012), extending the development of (social) cognition to include issues apparently unrelated like active aging, early determinants of health and risk factors.

Physical and mental health is affected by a complex interplay of individual and social factors at personal, family, community, and national levels (Viner et al., 2012), as well as by individual differences in cognitive abilities (e.g., Romer et al., 2011), attachment history (Bowlby, 1988), and personality traits. As we are discussing throughout this paper, all these factors undergo dramatic modifications during adolescence that tend to slow down and stabilize as the individual becomes an adult—better yet, that adultness begins when these factors begin to slow down and stabilize. Given this framework, it is all too obvious that physical and mental health “leaks” heavily from adolescence into adult life.

The onset of schizophrenia, for example, typically occurs in late adolescence or early adulthood (Häfner and an der Heiden, 1997; van Os and Kapur, 2009; WHO, 2015). Also, the incidence of mood and anxiety-related dysfunctions increases during adolescence (Hankin and Abramson, 2001; Costello et al., 2002).

Adolescence, however, also presents risks and disorders of its own. For example, during this age individuals are probably the fastest, the strongest and the most resistant to disease that they will ever be; at the same time, their chances of dying from putting themselves at risk—through aggression, crime, promiscuity, reckless driving, and drug use—also peak. It follows that precisely such behaviors are the first cause of death in this age group (Casey and Caudle, 2013).

Risk behaviors appear to be caused by diminished self-control, sensation-seeking behaviors and peer pressure. Their determinants are frequently searched in the brain development and neuronal connections (e.g., Steinberg, 2008; Telzer et al., 2013). Several authors have also found a peak in risk taking as evaluated by laboratory tasks involving emotions (Figner et al., 2009; Cauffman et al., 2010) and decision making (Wolf et al., 2013).

A better understanding of adolescence and its features in terms of social cognition would thus have profound implications for protection and prevention. To relate scientific researches and methodologies to real-life issues appears to be crucial in the study of adolescence. Yet, a more critical analysis of the frequency and the contexts of occurrence of risk-taking behaviors in adolescence (Willoughby et al., 2013), an articulated model to understand the evolutionary functions of adolescence (Ellis et al., 2012), and a sound theoretical framework for social cognition during this age, are still needed to complete the picture (and then to develop its implications for protection and prevention).

## What is Adolescence?

The rough definition of an adolescent as an individual who is no longer a child but not yet a true adult might seem poor and fuzzy from a scientific point of view, but it is probably the most effective in capturing the complexity and contextual dependence of the phenomenon called adolescence (e.g., Moshman, 2005; Hopkins, 2014).

In discussing adolescence, indeed, not only do we have to consider the high variability between individuals, but we also have to take into account that different societies define adolescence in terms of ages and social roles with comparatively little consistency (Sawyer et al., 2012). Furthermore, most societies throughout human history have not acknowledged the existence of an age called “adolescence,” at least as we understand it (e.g., Kett, 1977; Hine, 1999; Hopkins, 2014). Many of the 1.8 billion youngsters mentioned above are likely not, or not fully, considered adolescents within their social contexts.

The beginning of adolescence is commonly identified with puberty, that is a complex biological transition which is universal in the human species, although the age at which it occurs may vary depending on features of both the individual and the context.

The decrease in the age of puberty onset that has taken place since the twentieth century in high-income countries (Sawyer et al., 2012) demonstrates the role of contexts in shaping individual trajectories, whereby the improvement in economic and material conditions like childhood hygiene, nutrition, and health appears to play a major role. At the same time, in those countries, sociocultural conditions like a longer education, possible delays or difficulties in employment, late marriage and childbearing have extended the duration of adolescence and changed its shape (Sawyer et al., 2012).

Adolescence looks different when viewed from one boundary or the other: basically, its beginning depends on biological and material features of the context, while its end depends on cultural and social factors. This also entails that individual differences become more important with age, while the merely chronological data become less important.

Around and with puberty begins a multidimensional and multilayered dynamics that involves every aspect of the individual’s life. The young members of several cultures undergo specific rites of passage that take puberty as the symbolic threshold beyond which a child becomes an adult. Many cultures set one or more later thresholds after which the individual will be legally considered an adult as far as her rights and duties of a political, juridical, military, work and otherwise formal nature are concerned.

However, these further thresholds are merely relevant to legal and social norms, so much so that Black’s (1990) law dictionary defines adolescence as “the age which follows puberty and precedes the age of majority.” Thus, an adult is anyone whom the state legislation says is an adult, by modifying the permissions, obligations and prohibitions that in different ways change her social contexts and spheres of interaction as a member of the community. However, there is no reason to think that these norms depend on clear changes in the individual’s cognition, whether social or otherwise.

A chronologically based definition promotes an understanding, typical of Western societies, of adulthood as taking place within the individual, with no concern for the social context: the end of adolescence is determined by law, with exclusive reference to age and not to the individual’s interaction with other people and events (Schwartz et al., 2010). We opt for an operational definition of adolescence, instead of a legal/chronological one. Of course, this might turn out to be hardly manageable at the empirical level, where researchers need to have well-defined groups of subjects available; yet, we think that, at least on the theoretical level, this is a more sensible approach.

In fact, there may be different formal threshold ages for the different facets of citizenship, and they may vary from nation to nation or from decade to decade according to the historical context.

In other words, there is no biological threshold after puberty as well as no psychological threshold in a strict sense: the body, including the brain, progressively changes until it slowly reaches a mature stage, but it cannot be said to achieve a literally steady state, nor a state which be clearly distinguishable from adolescence. Its development never really ceases: it just slows down after the fast-paced events ongoing during adolescence proper. The same, of course, holds for cognition in general, and for social cognition specifically.

This makes identifying an end to adolescence a particularly difficult task. A phrase like *coming to terms with adulthood* is vague enough to warrant widely (or even wildly) different interpretations; and anyway, as we have said, adulthood may be differently conceived of in historically different contexts or even in different domains of an individual’s life. Thus, for example, in many an affluent country it is comparatively normal to witness a divergence between the age at which an individual is legally and psychologically capable of living an autonomous adult’s life and the individual or contextual conditions—like a particularly prolonged education, or the prices of housing and living—that may make this socially or subjectively unaffordable (Sawyer et al., 2012). In other countries or in other socioeconomic situations, of course, things may be hugely different: think, for example, of the many areas, in affluent as well as in less protected environments, where youngsters may legally or illegally be employed or exploited as slave workers, prostitutes, soldiers, or even suicide bombers. In this sense adolescence may be viewed as socially constructed by a society that can and wants to afford it.

A definition of adolescence as *coming to terms with adulthood* may appear to imply that mental life during this age should be viewed as a precursor to that of full adulthood and thus exclusively or prevailingly understood in terms of the latter: the adolescent would then be nothing more than a “future adult.” This merits a brief discussion.

There are indeed various ways in which a sort of teleology belongs to this framework. At least some of the environments in which the adolescent participates are explicitly or implicitly conceived and structured so to offer the cognitive, social and cultural tools that will help her to acquire the knowledge, the competencies and the other skills that will be required of her as an adult: schools, professional education, reformatories and military academies are, of course, the most visible, important and formally structured environments of this kind, but there may be others as well. These environments in which the adolescent participates require her to think and act like an adult: many such contexts will be tolerant of “adolescent behavior,” but others will not, or not completely. So far, we have a sort of a later-age, probably less tolerant, equivalent of Vygotsky’s notion of the Zone of Proximal Development (Vygotsky, 1978; Daniels et al., 2007).

On the other hand, it should be noted that the opposite process may also be at work, whereby typically in the affluent areas of the world adolescents are *teenagers*, that is a specific market segment with features of its own. Of course, this also requires selling the idea that adolescents are *not* adults, that they have a mental and social functioning all of their own, and so on. The coexistence of these two processes increases the complexity of the adolescent’s mental and social life.

Furthermore, the adolescent represents herself at least partially in accordance with the psychological, social, and cultural coordinates in which she finds herself. For the first time, she entertains a visible temporal and ontogenic horizon which she may strive to achieve; that is, she represents her own future in a non-oneiric way and may make concrete choices that are, or at least aim to be, consequential. She thus oscillates between two centers of gravity. One such center is the awareness and the expectation that she is soon going to be an adult: she has an idea, however approximate, of what this means, and might work toward such end, e.g., by going to school or by learning the skills that (she thinks) will help her reach her goals as an adult. The other center of gravity is the fears and the other emotions that prospective adultness may ingenerate, and the desire to enjoy the space of freedom which society allows her, in the fear of losing it with passing years.

Thus, teleology in adolescence is different from what it could be during childhood. While in both periods the individual needs be considered in her present time, which she lives with the full autonomy that her age allows for, adolescence is characterized by internal as well as external pressures and tensions between such present and a future which is both the object of representations, expectations and positive and negative feelings, and a set of cognitive, social and cultural tools that offer a scaffolding (again in a Vygotskian sense) which the individual has available to govern her own future, in a circularity whose features are those of a partially self-fulfilling prophecy.

Summing up, adolescence begins with puberty and is characterized by the intertwining of several kinds of changes:

(i) biological and psychological changes that are universal, albeit occurring at different ages;
(ii) socio-cultural and psychological changes that are practically universal, at least in their abstract or symbolical form, called rites of passage;
(iii) socio-cultural and psychological changes that are local and contextual, varying from the status of a young soldier, slave or prostitute to that of a “teenager” in an affluent family;
(iv) the appearance of true autonomy and self-government, with a still budding ability to deal with them and their internal and external sources and consequences.

Adolescence has no precisely identifiable end; it slowly shifts into adulthood as the individual comes to terms with his new state. *Coming to terms with adulthood* may have many different meanings, depending on the groups and the society in which an individual finds himself, of the requests posed by his environment, the resources and the opportunities he finds available, and so on. While puberty is a necessary biological and psychological transition, adulthood is defined contextually; in certain contexts a reasonable level of adultness may be reached at different ages in different domains, and often many years after biological maturity, while in others a distorted adultness ends up to be superimposed on an individual who is not yet ready to cope with the ensuing set of activities and interactions. Each of this paths may be laden with problems and tensions, which may then relapse on the individual’s psychological or social wellbeing.

## What Adolescence Requires of Social Cognition

From the subjective point of view, at least in Western countries, adolescence is a time when an individual finds herself confronting the feeling, simultaneously endogenous and exogenous, of becoming an adult. When she has become reasonably able to come to terms with these feelings, this phase ends; or, better, it shifts seamlessly into “full adulthood.”

To conceive of adolescence as the beginning of adulthood brings one to also view social cognition during this stage of life as remarkably different from what it used to be during (the various phases of) childhood and more akin to what it will be at later ages. So, what developments in an individual’s social cognition characterize this phase?

Changes in social cognition during adolescence are both inward and outward. The latter may be summarized as a further opening toward the world.

Several studies show how social networks change across the life span. Interactions during infancy and childhood normally take place within the family or in quasi-familiar environments like primary school. The global social network grows during adolescence, when the individual gains emotional and behavioral autonomy from parents, and then tends to decrease throughout adulthood (for a meta-analysis, see Wrzus et al., 2013).

An adolescent’s social network becomes wider but also more impersonal in some of its zones. Adolescents are involved in situations that require them to take a role in a strict sense, including, e.g., secondary school, working places and other social grounds characterized by formal and formally accountable expectations and behaviors. The types of norms that this requires them to handle are not, or not mainly, behavioral rules in the form of do’s and don’ts like those that are learned during childhood; instead, they are complex systems of social positioning and reasoning that establish a certain worldview from which situated social actions should be derived moment by moment. Things are even more complicated, and more challenging for social cognition, insofar as formality often is only part of the situation, a sort of facade that belies complex informal interpersonal, social, and (largely speaking) political dynamics.

On a still more abstract level, this new conception of social worldviews, social grounds, and social roles typically expands to system-wide dynamics. The adolescent begins to interact with and within the society at large and its structures and institutions, e.g., in terms of understanding and dealing with citizenship, or of getting intellectually interested or materially involved in local, national or international politics or economics. This is the age when an individual may begin reading the news, taking a political stance, participating in demonstrations or, in more troubled contexts, more or less voluntarily carrying a weapon in a war. Actually, possibly fueled by a still partial understanding of the relevant dynamics and of their visible and hidden complexities, the civic and political fervor of this period will seldom be found again at later ages. Recent approaches to youth health and development have taken a turn from the traditional view of youths as victims or problems of society and passive recipients of adult-directed interventions to one that portrays them as powerful catalysts to community change by acting as resources and competent citizens in their communities (Makhoul et al., 2012).

We argue that these interests and activities fully engage social cognition insofar as they require understanding social habits and norms, the ways in which different individuals may follow, exploit or violate such norms, the positions that different individuals occupy in groups, organizations and the society at large, how they have reached there and how they tend to interpret such position, and so on.

Within areas more classically acknowledged as relevant to social cognition proper there are at least two other crucial realms that need be considered about social life in adolescence, namely interaction with peers and interaction with romantic or sexual partners. While neither of them appears, strictly speaking, in adolescence, both take on whole new nature and roles with respect to what they used to be in childhood.

Peers may happen to be other individuals, different groups of which the adolescent is or is not a member (including allied or various rival or antagonistic groups), other organizations of which she is or is not a member, and so on. In a different acceptation of interaction, peers may even be the *representations* of the peers that are projected by the various social and cultural contexts in which the adolescent participates, like her family, her friends, the media, or the society at large. Such projections may be descriptive or normative in different ways, ranging from the rules and expectations that are enforced by the family or the group of peers to narratives found in books and movies, marketing and advertising in the media, and so on.

Interactions with peers, like all interactions, require the adolescent to handle habits and norms, hierarchies and statuses, and to understand how arrays of other individuals do the same. At the same time, however, these interactions may be “hotter” than others to adolescents: they are less abstract, more situated, and more emotionally and intellectually compelling. Therefore, these immediate social dynamics cannot be handled with the tendentially formal rules with which the more removed ones discussed above are: dealing with peers thus tends to be *subjectively* more complex, more challenging, and more potentially awkward.

At least in several human societies, even more so may be the other crucial type of interaction that characterizes adolescence, namely the romantic and/or sexual, which may (at least in principle) address the individual toward the construction of a long-lasting relationship. This poses several problems. Courting someone, or letting someone court us, requires a complicated game of showing and concealing one’s feelings and intentions. Even more importantly, it requires recognizing, labeling, and wording one’s own feelings and emotions (like, “what is it that I feel? Is this what love is? or is it sexual desire?” and so on) as well as those of the partner’s. All of this turmoil may often involve further persons on one or the other side of the actual or potential couple, giving rise to even more complicated problems about cheating, jealousy, rejection, and so on.

Even such a brief outline of the changes that characterize outward social life and social cognition during adolescence makes it clear why an analogously complex change in inward, that is reflective, social cognition is needed. Basically, the adolescent has to devise cognitive tools and ways to deal, on the one hand, with all that is happening in the highly complex, multidimensional space of her outbound social life and, on the other hand, with her own rapidly changing personal identity.

This requires her, among other things, to be able to monitor, understand, explain, predict, abstract from, and, first and foremost, *feel*, her own mental dynamics: the kind of questions she has to answer may take forms like “what is this that I feel? whence do these thoughts and emotions come? how do I explain them? where do they lead me? how do I judge them? are they good or are they bad? how do I control them? how do I share them with other individuals, and how should I choose these individuals?”.

Internal tensions concerning this dynamics may easily ensue, due to the sheer difficulty of dealing with such complex questions, accepting their consequences for oneself and for various other persons, and accepting the continuous redefinition of personal identity that they propose. It is in this age that social dynamics as diverse as shame, pride, isolation, rage, rebellion, leadership and others thrive: in several senses, this is the end of innocence. It also comes as no surprise that adolescents are more at risk of deviant behavior as well as of becoming victims of hoaxes, deceits, and so on.

Summing up, an individual’s social cognition during adolescence is asked to deal in increasingly complex ways with (and, circularly, her social cognition begins to provide her with the ability of dealing with) different types of contexts:

(i) her own mind;
(ii) other, specific individuals (family, friends, colleagues or classmates, romantic or sexual partners, and so on);
(iii) other, generic individuals (strangers);
(iv) groups and organizations and their individual members acting as such.

This requires an intertwining of social cognition with other “cognitive functions” like planning and organizing one’s own actions and recognizing how others plan and organize theirs, processes of education, cultivation, and acculturation, an appropriate management of autobiographical memory, and so on.

## Data From Developmental Psychology

ToM is generally considered a crucial part of social cognition and has been extensively studied in developmental psychology.

Children’s ability to understand and reason about mental states has traditionally been investigated by testing their accuracy on mentalizing tasks, often based on false beliefs (Dennett, 1978), which are typically passed by 3- or 4-years-olds (Wimmer and Perner, 1983; Wellman and Liu, 2004). As discussed above, these kinds of tests rapidly reach a ceiling effect as the subjects’ age increases; a gap thus emerges in the literature after the pre-scholar period. Researches with children older than about four have used more complex ToM tasks (e.g., Happé, 1994; Baron-Cohen et al., 1999); however, precisely these methodological differences make it difficult to highlight continuities or discontinuities in development (Apperly et al., 2011).

In any case, it is hard to imagine that social cognition would not change with adolescence, if only because the individual’s general cognitive abilities change, as well as her social experiences do (Blakemore and Choudhury, 2006). In comparison with the large amount of researches investigating mentalizing abilities during childhood, however, only few studies explored the development of these capacities in adolescence and their relations with other life-span developments (Colvert et al., 2008; Apperly et al., 2011; Harenski et al., 2012).

The ability to reason about the mental states of the others and to understand and take into consideration what they think, feel and believe appears to require the ability to take another individual’s perspective, which in turn is crucial to successfully manage social communication. Perspective taking is related to first-order ToM, since it involves what another person is thinking; also, it requires the awareness of one’s own mental states (first-person perspective) and the ability to ascribe mental states to other individuals (third-person perspective; Blakemore and Choudhury, 2006).

In a study by Choudhury et al. (2006), children, adolescents and adults were tested with a perspective-taking task requiring them to imagine which emotion they themselves (first-person perspective) or another person (third-person perspective) would feel in different scenarios. The results showed that the differences in reaction time between first- and third-person perspective-taking decreased with age, suggesting that proficiency at perspective-taking improves between childhood and adulthood.

Dumontheil et al. (2010) tested the ability of a large sample of children, adolescents and young adults (aged 7–27) to use information received about another person’s point of view in a perspective-taking communicative task. Again, the ability to take another person’s perspective into account turned out to grow from infancy through adolescence with further improvements in adulthood.

Fett et al. (2014) showed that a greater inclination to take the others’ perspectives into account was associated with a stronger pro-social approach toward others and a stronger trust during cooperative interactions. In interactions with an unfair partner, this inclination was associated with a more drastic decrease of trust and less benevolent reciprocity.

Bosco et al. (2014b) assessed the ability to understand and manage mental states in pre-adolescence and adolescence using the ToM Assessment Scale (Th.o.m.a.s.: Bosco et al., 2009a; see also Laghi et al., 2014) and some well-known ToM tasks (namely a subset of the Strange Stories by Happé, 1994). Th.o.m.a.s. is a semi-structured interview organized along four scales, each focusing on one of the knowledge domains in which a person’s ToM may manifest itself; it provides a detailed profile of different facets of ToM abilities, namely first- vs. second-order, first- vs. third-person, egocentric vs. allocentric perspective. It also explores different types of mental states involved in ToM, namely beliefs, desires, positive emotions, and negative emotions. The results were that the performance at Th.o.m.a.s. improves with age, confirming that the ontogeny of ToM continues at least through adolescence.

Furthermore, in agreement with Goldman’s (1993) hypothesis that adolescents can better reason about their own mental states than about those of the others, the participants performed better at first-person than at third-person tasks. This appears consistent with the widely diffused perception that a typical feature of preadolescence and adolescence is a tighter focus on the attempt to understand oneself than the others. Also, again in agreement with previous literature (Wellman and Liu, 2004), the adolescents performed better at first-order than at second-order ToM tasks. No significant difference emerged, instead, between the allocentric and the egocentric viewpoint.

The performance at Strange Stories did not reveal any significant age-related difference; however, there is no evidence in the literature that there should be any. While Strange Stories are considered advanced ToM tasks, they were originally developed for children (Baron-Cohen, 1989; Happé, 1994) and thus they too are probably unfit for the study of mentalization at later ages.

Th.o.m.a.s. also investigates the ability to deal with different types of mental states (beliefs, desires, positive emotions, negative emotions). In Bosco et al. (2014b), the participants scored higher at negative emotions than at the others, a result that could be explained with the turbulent psychological and relational changes that characterize adolescence, together with a sort of existential confusion which is likely to lead a person to reflect more deeply on her own negative emotions.

The ability to make inferences about emotions is called *affective ToM* and may be conceptualized as the integration of *cognitive ToM* (inferences about knowledge and beliefs) and empathy (Shamay-Tsoory et al., 2010). In a study by Sebastian et al. (2011), adolescents made more errors than adults in choosing the appropriate ending of vignettes depicting a character’s response to a companion’s emotions. Vetter et al. (2013) used film clips depicting the manifestation of emotions to investigate affective ToM across adolescence, finding that it develops with age. They also found that it correlates with executive functions (specifically inhibition) throughout adolescence until young adulthood.

The phrase *executive functions* refers to the cognitive processes involved in goal-directed actions, such as those that allow an individual to control and coordinate his thoughts and behavior (Shallice, 1982). These are, for example, selective attention, working memory, decision-making, and inhibition. Several behavioral studies show that the ability to manage tasks like inhibitory control (Leon-Carrion et al., 2004), processing speed (Luna et al., 2004), working memory and decision-making (Hooper et al., 2004) continues to develop during adolescence. A correlation between affective ToM and inhibition has also been found in studies conducted with young adults (Bull et al., 2008; Ahmed and Miller, 2011). In pre-school children, executive functions have been shown to play an important role in ToM performance (Carlson and Moses, 2001), which suggests that they may play an important role in the subsequent developmental stages as well.

Another construct which is commonly held to be closely related to social cognition is metacognition, defined as the ability to think about thinking (Flavell, 1979). Semerari et al. (2003, 2007) view it as composed of an array of independent subfunctions that concur to its overall functioning, namely the abilities to recognize one’s own thoughts and emotions, to relate them to the relevant interpersonal events, to understand the mental states of other persons and to keep them distinct from (and possibly different to) those of one’s own, to acknowledge that mental states incorporate a point of view and therefore are fallible, to describe mental states modifications in a coherent narrative, and to control and adjust internal states (*mastery*: more about this later). Based on this theoretical elaboration, Semerari et al. (2012) built and validated a semi-structured interview called the Metacognition Assessment Interview (MAI), that has been administered to persons with schizophrenia or personality disorders. What is relevant to our current purposes is the notions that social cognition is a highly complex faculty, far from being reducible to simpler processes, even if complex in their own way like ToM, and that there can be individual or ontogenetic differences in the persons’ capacity to handle it.

Metacognitive mastery has been correlated with quality of life (Lysaker et al., 2005) and the complexity of social functioning (Lysaker et al., 2010) in persons with schizophrenia. In general, metacognition appears to be able to influence several aspects of experience (Metcalfe, 1996) such as self-regulating learning (Efklides, 2009) and decision making (Weil et al., 2013). An impairment in the ability to reflect on and to use knowledge about the mental states of one’s own and those of the others may hamper the ability to cope with complex psychological and relational challenges and thus lead to dysfunctional reactions to individual or interactional difficulties (Lysaker et al., 2010). Given the dramatic number of new experiences and transformations characterizing adolescence, these issues and their interplay may end up playing a significant role.

Demetriou and Bakracevic (2009) investigated metacognitive ability asking adolescents and adults to assess their own performance on propositional, spatial and social reasoning tasks; this self-evaluation was found to improve from adolescence to adulthood.

Weil et al. (2013) analyzed the development of metacognitive ability from adolescence to adulthood, during a visual task implying decision-making processes. Their results showed that the awareness of one’s own perceptual decisions undergoes a prolonged developmental trajectory during adolescence, again suggesting that metacognitive ability significantly improves with age.

## Somatic Changes and Brain Maturation

Adolescence brings with itself a vast array of bodily modifications. Some of these modifications, namely those in the brain, are not immediately available to the subject’s awareness, while others are external and dramatically important from a subjective point of view.

Adolescence, for example, normally entails a sudden, rapid, and remarkable upgrowth. One finds herself watching the world from about the same level from which adults watch it, sometimes from an even higher one. This alone will obviously yield a difference in how one perceives social relations, hierarchies and statuses, power (whether social or crudely physical), dependability, and so on. Perspective taking here is laden with an experiential burden that makes it impossible to reduce it to a “cognitive” issue in the classic acceptation of such word. The growth of muscle mass and the increase in agility and physical strength add to this change of one’s body image.

Even more the same holds for sex-related morphological changes, like the development of primary and secondary sex characteristics. To find oneself being the agent or the recipient of sexual attention, sexual desire and sex-related activities, as well as *not* being one despite one’s desires or needs, has an obvious impact on one’s social attitudes. To acquire and to come to terms with the relevant set of thoughts, feelings, emotions, habits and so on, both in oneself and in the others, is a seemingly impossible task that, nevertheless, needs be accomplished with reasonable speed and efficiency.

A completely different set of data and considerations is supplied by other, more classic areas of research.

Several authors suggest that the endocrine changes that characterize puberty influence brain development and restructuring during adolescence (Sowell et al., 2002; Lenroot et al., 2007; for reviews, see Peper et al., 2011; Peper and Dahl, 2013). Functional magnetic resonance imaging (fMRI) studies provide evidence of the plasticity of the adolescent’s brain, characterized by a general structural development, a synaptic neuronal reorganization and increase in connectivity (Sowell et al., 2003). Social cognition, particularly the ability to mentalize, is associated with a network of brain regions commonly referred to as the “social brain” (Frith and Frith, 2003). This network appears to be the counterpart of the ability to recognize other persons’ mental states like intentions, feelings, emotions, desires and beliefs, and to use such recognition to understand their behavior. The social brain includes several areas: the medial prefrontal cortex (mPFC), the anterior cingulate cortex (ACC), the inferior frontal gyrus, the superior temporal sulcus (STS), the amygdala and the anterior insula (Blakemore, 2008).

Several studies offer empirical evidence of the development of the social brain during the adolescence (for a review, see Blakemore, 2008). Recently this subject has received renewed interest. Mills et al. (2014) studied the structural development of the social brain from late childhood through adolescence and into adulthood. They found that gray matter volume and cortical thickness in the mPFC, the temporoparietal junction and the posterior STS decreased from childhood into the early twenties. The anterior temporal cortex increased in gray matter volume until adolescence and in cortical thickness until early adulthood. The surface area for each region peaks in pre-adolescence or early adolescence before decreasing into the early twenties. The authors suggested that the reductions in gray matter volume may reflect synaptic reorganization and concluded that the social brain network continues to develop structurally across adolescence before relatively stabilizing in the early twenties.

Klapwijk et al. (2013) analyzed the relations of a set of endocrine and somatic pubertal indicators with functional connectivity in the social brain (dorsomedial prefrontal cortex, right posterior STS and right temporoparietal junction) involved in emotion processing in girls aged 11–13 years. Their results suggest that the progress of puberty in girls is related to the functional maturation of the social brain.

Finally, Goddings et al. (2014) found that puberty has a important role in influencing the subcortical development of the brain. They analyzed data from longitudinal magnetic resonance imaging scans of individuals aged 7–20 years, finding an interactive puberty-by-age effect on the volume of the nucleus accumbens, the globus pallidus and the caudate: these regions are involved in reward-seeking behaviors and decision-making processes (Gottfried, 2011). Goddings et al. (2014) also found pubertal effects on the growth of the amygdala, which is involved in emotion processing. Pubertal changes include modifications in the neurobiology of stress and emotions, capable of shaping reactivity to stressors and affective stimuli (Spear, 2009). These modifications may precipitate the emergence of psychopathologies in vulnerable individuals and contribute to the emergence of psychological disorders (Dahl, 2004; Walker et al., 2004).

Globally considered, these modifications appear to be related to several aspects of mind functioning that characterize social cognitive ability (see also Moriguchi et al., 2007). It probably is no coincidence that many of the functions whose cerebral counterparts undergo such modifications are those in which adolescence looks more stormy and potentially unsafe. Yet, brain studies can hardly link brain modifications to a person’s social and cultural experience. In this sense, the “social brain” should be viewed and studied as a feature not of the individual, but of the contexts in which the embedded, situated, and embodied individual participates.

## Conclusion

Adolescence is an extremely interesting as well as challenging topic for the study of social cognition.

A first issue is that context-free studies can hardly be devised. The social, cultural, educational, economic, and autobiographical situations in which the individuals participate play too important a role in how they experience and enact their social life. For the same reason, it is likely impossible to devise empirical methodologies similar to those that are commonly employed in the study of other aspects of the functioning of the mind or of the social life in infancy and childhood. Not coincidentally, a comparatively advanced ToM task like the Strange Stories (Happé, 1994), differently to the false-belief tasks that are used with children, is built around a narrative infrastructure and its contents are culturally localized.

A second issue is that the empirical methodologies should be adjusted to keep into account the vast differences in social situations, cognition, and actions that exist between different contexts, between different individuals, and between the different domains and activities in which the same individual may participate.

A third issue is that the notions that are generally employed for the study of social cognition, like ToM, are probably insufficient to account for it. In a phenomenological approach such as that adopted, for example, by Gallagher (2006), the notion of a ratiomorphic, purely inferential ToM appears to be far too simplistic to account for the richness and the complexity of human social experience. The alternative proposed by Gallagher is in terms of a narrative competence, which in its turn would be grounded in the direct perceptual access to the intentions and the feelings of others: in this approach, second-person interactions would replace the more typically studied observations in the third-person; and, of course, the situated, embedded, embodied, autobiographically rich first person would be the center of gravity of the whole narrative.

Theory of mind would then intervene when a breakdown occurs, analogously to how theories in naïve (or non-naïve) physics intervene when our bodily experience, normally grounded in habits and choreographies, encounters a breakdown.

Thus, according to Gallagher and Hutto (2008), it is not the inner life or the mental life of the others that we attempt to access, but their life in its worldly contexts, which is best captured in a narrative form. Life events, including social interactions, happen as stories with a beginning, a development, and possibly an end, that take place in the world and in which we, and the others, may happen to play a part (see also Goffman, 1959).

Adolescence may then be viewed as an age in which the narratives change suddenly and profoundly, opening a world of new possibilities, new promises, new dangers. Cast abruptly in this new world, the adolescent has to wade through it, finding her own way to deal with the new situations in which she wants and needs to find herself. In this task, social life is simultaneously a huge source of problems, opportunities, and resources. That most of us survive this storm to find comparatively calmer waters is one of the most amazing feat of human kind.

### Conflict of Interest Statement

The Reviewer Tuula M. Hurtig declares that, despite being affiliated to the same institution as the author Ilaria Gabbatore, the review process was handled objectively and no conflict of interest exists. The authors declare that the research was conducted in the absence of any commercial or financial relationships that could be construed as a potential conflict of interest.

## Footnotes

1 Throughout the paper we will use either the feminine or the masculine when referring to unspecified human beings. This is only made for the sake of readability and is not meant to convey any gender-related position or the idea that there may or may not be differences in social cognition between genders. However important, such themes are simply not relevant on the level of abstraction on which our arguments are cast.
